# The Effect of Imbalanced *Progesterone Receptor-A/-B* Ratio on Gelatinase Expressions in Endometriosis

**DOI:** 10.22074/ijfs.2019.5604

**Published:** 2019-04-27

**Authors:** Sepideh Mousazadeh, Azadeh Ghaheri, Maryam Shahhoseini, Reza Aflatoonian, Parvaneh Afsharian

**Affiliations:** 1 Department of Genetics, School of Natural Sciences, University of Tabriz, Tabriz, Iran; 2 Department of Genetics, Reproductive Biomedicine Research Center, Royan Institute for Reproductive Biomedicine, ACECR, Tehran, Iran; 3 Department of Epidemiology and Reproductive Health, Reproductive Epidemiology Research Center, Royan Institute for Reproduc- tive Biomedicine, ACECR, Tehran, Iran; 4 Department of Endocrinology and Female Infertility, Reproductive Biomedicine Research Center, Royan Institute for Reproductive Biomedicine, ACECR, Tehran, Iran

**Keywords:** Endometriosis, Gelatinases, Progesterone, Progesterone Receptor

## Abstract

**Background:**

Gelatinases degrade extracellular matrix (ECM) components to allow for physiological remodeling and contribute to pathological tissue destruction in endometriosis. It is known that the function of gelatinases is resistant to suppression by progesterone in endometriosis. The ability of progesterone to impact gene expression depends on the *progesterone receptor-A/-B (PR-A/PR-B)* ratio. An imbalanced *PR-A/PR-B* ratio in endometriotic tissue may be the result of the differential expression of *MMP-2* and *MMP-9*, which could be important in the etiology and pathogenesis of the disease. Hence, we decided to study the association of *PR-A/PR-B* ratio and gelatinases expression in endometriosis.

**Materials and Methods:**

In this prospective case-control study, we enrolled 40 women, 20 in the case group who were diagnosed with stage III/IV endometriosis and 20 normal subjects without endometriosis (controls) who referred to Royan Institute, Tehran, Iran during 2013-2014. We obtained 60 tissue samples [ectopic (n=20), eutopic (n=20), and normal endometrium (n=20)]. RNA was extracted from the tissue samples in order to analyze *PR-A, PR-B, MMP-2,* and *MMP-9* mRNA levels through real-time polymerase chain reaction (PCR).

**Results:**

There was significantly lower expression of the *PR-B* isoform in ectopic tissues compared to the control (P=0.002) and eutopic endometrium (P=0.006) tissues. *PR-A* expression was higher, but not significantly so, in the same ectopic and eutopic endometrium tissues compared to the control tissues (P=0.643). There was significant over- expression of *MMP-9* in ectopic samples compared to control (P=0.014) and eutopic endometrium (P=0.012) samples. The *PR-A/PR-B* ratio was not significantly higher in either eutopic or ectopic samples compared to the control samples (P=0.305).

**Conclusion:**

Our findings support an altered *PR-B* expression in endometriosis, which may be associated with *MMP-9* overexpression. This finding can be important for disease pathogenesis.

## Introduction

Infertility is a persistent and frustrating problem in women with endometriosis (1). The frequency of endometriosis in females with complaints of pain, infertility, or both symptoms is between 35 and 60% (2). It is suggested that endometriosis affects the follicular microenvironment, oocyte maturity and embryo development (1, 3). Extensive remodeling in the endometrial layer and its extracellular matrix (ECM) is one of the reasons for infertility in endometriosis (1, 4, 5). This remodeling of the ECM is required for the activation of matrix metalloproteinases (MMPs) and their inhibitors (6). The decreased potential for embryo implantation is thought to be one of the critical reason for infertility in women with this disease (1). High concentrations of activated macrophages, prostaglandins, IL-1, TNF, and proteases have been reported in peritoneal fluid of women with endometriosis. These abnormalities may adversely impact oocyte function, embryo development, and implantation (4).

MMPs or Matrixins are calcium/zinc-dependent endoproteinases encoded by 24 distinct genes and expressed as 26 distinct proteins in humans (7). They are secreted in a latent form (pro-MMPs) that require proteolytic activation (8). The biological roles of MMPs are associated with degradation of the ECM to provide normal endometrial remodeling that accompanies menstruation (9), proliferation, angiogenesis, and apoptosis (7). Endogenous tissue inhibitors of MMPs (TIMPs) regulate MMPs under physiological conditions such as tissue repair and menstruation (10-12). Numerous studies have discussed the role of endogenous proteolytic MMPs in the pathogenesis of endometriosis (13) and have reported a significantly different pattern of MMP expression in endometriosis patients compared to healthy women (14, 15). Over-expressions of MMPs alter the MMPs/TIMPs ratio that may underlie the pathogenesis of diseases including tumor invasion, fibrosis, and endometriosis (8, 16-18). MMPs are involved in all steps of endometriotic tissue migration such as degradation, invasion, and implantation to the ECM outside of the uterine cavity (19). Proteolytic enzymes, like gelatinases (MMP-2 and MMP-9), play an important role in the initial development of endometriosis through ECM degradation (20). The role of gelatinases in the development of diseases has been shown through the participation of MMP-2 and MMP-9 in tumor invasion and progression (1, 13).

Under normal conditions progesterone prevents endometrial breakdown by inhibiting MMPs (21) via its nuclear receptors (21, 22). However, in subjects with endometriosis there is a certain degree of resistance to the action of progesterone (23). In women with this condition, the eutopic endometrium is purportedly resistant to the action of progesterone and inhospitable for embryonic implantation (5). The effects of progesterone are controlled by the two progesterone receptor (PR) isoforms, namely PR-A (94 kDa) and PR-B (114 kDa). These isoforms are functionally different. The PR-B isoform is an activator of progesterone target genes, whereas PR-A is an inhibitor of the PR-B isoform (23). In addition, they are members of the superfamily of ligand-activated transcription factors that bind to sequence-specific sites in the promoters of target genes (22).

 On the other hand, progesterone represses *MMP-2* transcription in cells from the jar choriocarcinoma cell line by reducing PR and specificity protein 4 (SP4) through binding to the *MMP-2* promotor (24). Both overexpression and elevated activity of MMP-9 in endometriosis are believed to be regulated by nuclear factor kappa-B (NF-кB) (25). PR can directly interact with one of the subunits of NF-κB, RelA (p65) (26), which is necessary for NF-кB activation. Progesterone efficacy in gene expression depends on the ratio of *PR-A* to *PR-B* (27). An altered ratio in ectopic tissue might play an important role in the mechanism that causes progesterone resistance and modifies progesterone activity related to differential regulation of specific progesterone response genes, such as MMPs, which promote endometriosis. Greater understanding of the abnormal genetic mechanisms involved in the etiology and pathogenesis of endometriosis should lead to better diagnostic methods and targeted treatments that counter endometriosis and its symptoms.

## Materials and Methods

We conducted this prospective, case-control study in the Department of Genetics at Royan Institute, Tehran, Iran. Approval was achieved from the Institutional Research Ethics Board. The Ethics Committee of Royan Institute approved this study (No: EC/93/1047). All members signed an informed consent form prior to participation.

### Subject selection

This study was conducted from 2013 to 2014 at Royan Institute. We obtained 60 tissue samples (ectopic, eutopic, and normal endometrium) from 40 women. The case group comprised 20 patients with stages III and IV endometriosis. The control group consisted of 20 normal healthy women without endometriosis. Endometriotic (ectopic) tissues were collected during laparoscopy from all patients with ovarian endometriosis. The eutopic samples were obtained by pipelle sampling of endometrial tissues obtained from all patients. Endometrial samples from the control women were also obtained by pipelle sampling. The presence or absence of endometriosis was confirmed by laparoscopy and postoperative histology analyses in endometrial tissue samples from all study participants. Patients with confirmed diagnosis of endometriosis were placed in the patient group. Participants without endometriosis (normal tissue results) were assigned to the control group. None of the patients received hormonal treatments for at least 3 months prior to surgery and all reported regular menstrual cycles. Control group participants did not have any visible endometrial hyperplasia or neoplasia, infl ammatory or autoimmune diseases, or endometriosis at the time of the clinical examinations. We also confirmed that women in the control group had given birth to at least one child conceived through natural conception. The menstrual cycle phase at the time of surgery and biopsy was either during the proliferative phase (days 8-14) (80%) or secretory phase (20%) for both patients and controls.

### RNA extraction and cDNA preparation

RNA was extracted from snap-frozen tissue samples using TRIzol (Invitrogen, USA) according to the manufacturer’s instructions. Genomic DNA contamination was removed by RNase-free DNase I (#EN0521, Fermentas, Thermo Fisher Scientific, USA) and incubation at 37°C for 30 minutes. DNase I enzyme was inactivated by EDTA (50 mM, Fermentas, Thermo Fisher Scientific, USA) and incubation at 65°C for 7 minutes. cDNA samples were prepared from total RNA for each sample by one-step reverse transcriptase-polymerase chain reaction (RT-PCR) and a First-strand cDNA Synthesis Kit (K1632, Fermentas, Thermo Fisher Scientific, USA). Synthesized cDNA was stored at -20°C until later use.

### Quantitative real-time polymerase chain reaction

mRNA expression analysis was performed using SYBR^®^ Pre mix Ex Taq II (Applied Biosystems, USA) on a Lightcycler System, 7500 software version 2.0.1 (Applied Biosystems, USA) as recommended by the manufacturer. We used Primer 3 (version 4.0; http://primer3.ut.ee/), Gene Runner (version 3.05; www.generunner.net), and Perl Primer software (version v1.1.20; perlprimer.sourceforge.net) to design the specific primers used for amplification of *MMP-2, MMP-9, PR-A, PR-B,* and *β-actin* (internal control gene). These sequences were analyzed by Nucleotide Blast and Primer Blast in the NCBI database (http://blast.ncbi.nlm.nih.gov/). Table 1 lists the primers used in this current study and their expected product-sizes. Primers were purchased from Pishgam Co., Iran.

**Table 1:** Sequences of *β-actin, MMP-2, MMP-9, PR-A,* and *PR-B* primers


Name	Primer sequence (5ˊ-3ˊ)	PCR product (bp)

*β-actin*	F: CAAGATCATTGCTCCTCCTG	90
	R: ATCCACATCTGCTGGAAGG	
*MMP-2*	F: GCAACCTGTTTGTGCTGAAG	198
	R: GTAGCCAATGATCCTGTATGTG	
*MMP-9*	F: TCCAGTACCGAGAGAAAGCCTA	114
	R: GCAGGATGTCATAGGTCACG	
*PR-A*	F: AATGGAAGGGCAGCACAACT	192
	R: TGTGGGAGAGCAACAGCATC	
*PR-B*	F: AAGGGGAGTCCAGTCGTCAT	165
	R: CGAAACTTCAGGCAAGGTGT	


*MMP; Matrix metalloproteinase, PR; Progesterone receptor,* and PCR; Polymerase chain reaction.

Each reaction contained 10 μl SYBR^®^ Premix Ex Taq II that consisted of Taq DNA polymerase reaction buffer, dNTP mix, SYBR Green II, MgCl_2_ and Taq DNA polymerase; 5 pmol of either *MMP-9, PR-A,* or *PR-B* primers, or 3 pmol of *MMP-2* primer; 25 ng/μl of synthesized cDNA; and water to reach 20 μl. The target gene levels were compared to that of a housekeeping gene, *β-actin*, from the same cDNA. Each real-time quantitative PCR assay was done in duplicate for each sample to confirm the reproducibility of the results. In this study, both housekeeping genes *GAPDH* and *β-actin* were optimized; however, the expression of *β-actin* appeared to be more stable in our samples. The amplification program contained the following 3 steps. Step 1: a primary heating for 10 minutes at 95°C to denature the cDNA and activate the Taq DNA polymerase. Step 2: DNA amplification for 40 cycles of 15 seconds at 95°C (denaturation) and one minute at 60°C (annealing) for *β-actin, MMP-2, MMP-9, PR-A,* and *PR-B*. Step 3: increasing temperature gradually from 60°C to 95°C for 15 seconds and one minute at 60°C for melting curve analysis. After each run, a melting curve analysis was done to confirm the specificity of the PCR reaction. All samples were retested with a cycle threshold coefficient of variation value higher than one degree. To confirm the melting curve results, we assayed representative samples of the real-time PCR products on 2% ultra-pure agarose (Invitrogen, USA) gel electrophoresis (Paya Pazhoh Pars, Iran), and stained them with ethidium bromide (Sigma Aldrich, USA) prior to visualization on a Molecular Imager^®^ Gel Doc™ XR+ (BioRad, USA).

### Statistical analysis

We compared the participants’ clinical information between groups (endometriosis and control) using the independent t test. The expression levels of *MMP-2, MMP-9, PR-A,* and *PR-B* were compared between tissue extracts of endometriotic or ectopic lesions and eutopic endometrium samples (patient group) to endometrial samples (control group) using one-way analysis of variance (ANOVA) followed by Tukey’s test to conclude significant differences between our groups and pair-wise comparisons. In cases where the data were not distributed normally, we conducted natural logarithmic (Ln) transformation for *MMP-2, MMP-9, PR-A, PR-B,* and *PRA/PR-B* before analysis. The relationships between the Ln-transformed expressions of *PR-A* and *PR-B,* as well as the *PR-A/PR-B* ratio with *MMP-2* and *MMP-9* were assessed by Pearson’s correlation. Statistical analysis was done using SPSS version 16.0 (SPSS Inc., Chicago, IL, USA). All statistical tests were two-tailed and a P<0.05 was considered statistically significant.

## Results

Table 2 shows the main clinical characteristics of the 40 participants who provided tissue samples. All 20 women with endometriosis were infertile. There were no statistically significant differences in the sample distributions according to the phases of the menstrual cycles, mean age, or body mass index (BMI) in patients with endometriosis compared to the control group.

### Expression of *MMP-2* and *MMP-9* in endometriosis

**Fig 1:**
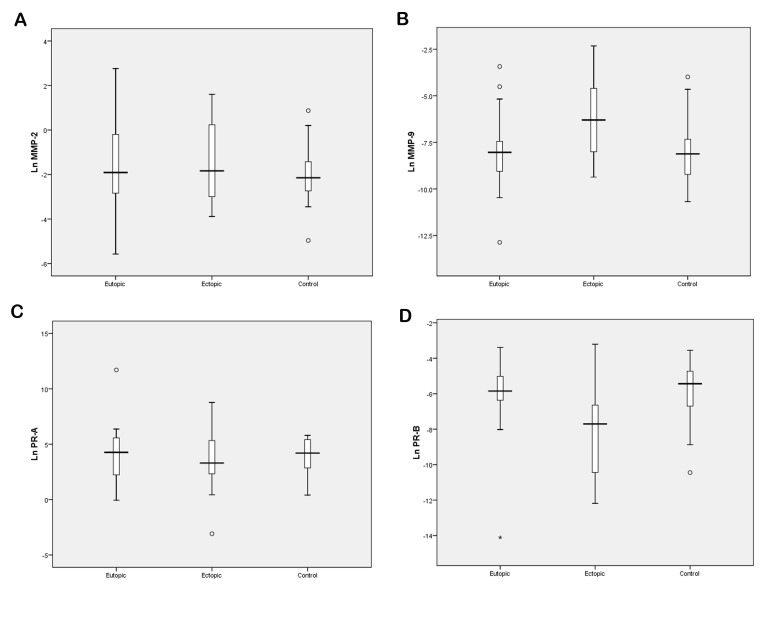
Expression levels of matrix metalloProteinases (*MMPs*) and progesterone receptors (PRs). **A.** *MMP-2*, **B.** *MMP-9*, **C.** *PR-A*, and **D.** *PR-B* in ovarian endometrioma (ectopic) and endometrial tissues from women with (eutopic) and without endometriosis (control). Ln: Logarithmic.

We assessed the differences between the means of mRNA levels in patients and controls with one-way ANOVA. We observed no significant difference in the expression levels of *MMP-2* among these groups (P>0.05, Table 3, Fig .1A). Our results showed a significant increase in the expression of *MMP-9* in endometriotic tissues compared to eutopic endometrium samples (P=0.012) and the control group (P=0.014, Table 3, Fig .1B).

**Table 2:** Clinical characteristics of participants in expression assays


Groups	Menstrual cycle phase (%)	Disease stage (%)	BMI (kg/m^2^)	Age (Y)

Endometriosis	Proliferative (80)	IV (60)	25.82 ± 4.91	30.03 ± 8.31
n=20	Secretory (20)	III (40)		
Controls	Proliferative (80)	-	24.35 ± 4.32	29.21 ± 8.72
n=20	Secretory (20)			
P value	NS	-	NS	NS


Data are expressed as mean ± SEM and values in parentheses are percentages. BMI; Body mass index and NS; Not significant.

**Table 3:** mRNA expression levels of *MMP-2, MMP-9, PR-A,* and *PR-B* in ovarian endometriosis and endometrial tissues obtained from women with and without endometriosis


Different object	Endometriotic lesions (ectopic)	Eutopic endometrium (endometriosis group)	Endometrium (control group)	P value

*MMP-2*	0.16 (0.05, 1.45)	0.15 (0.05, 0.87)	0.12 (0.06, 0.29)	0.512
*MMP-9*	0.02E-1 (0.03E-2, .021)^*Δ^	0.03E-2 (0.01E-2, 0.06E-2)	0.03E-2 (0.09E-3, 0.07E-2)	0.005
*PR-A*	27.20 (9.99, 206.33)	76.98 (9.00, 268.03)	67.05 (16.60, 231.78)	0.643
*PR-B*	0.04E-2 (0.03E-3, .01E-1)^*Δ^	0.03E-1 (0.01E-1, 0.07E-1)	0.04E-1 (0.01E-1, 0.09E-1)	0.001
Ln (*PR-A/PR-B*)	11.79 ± 4.82	10.28 ± 4.64	9.81 ± 2.93	0.305


Data are expressed as mean ± standard deviation or median (inter-quartile range) when appropriate. ANOVA was performed on the natural-log-transformed values when appropriate. *MMP*; Matrix metalloproteinase, PR; Progesterone receptor, *; P<0.05 versus endometriotic lesions compared to the controls, and ^Δ^; P<0.05 versus endometriotic lesions compared to the eutopic endometrium.

### Progesterone receptor isoforms *PR-A* and *PR-B* expression in endometriosis

Extracts of endometriotic lesions from women with endometriosis presented a slight decrease in mRNA level of *PR-A* in comparison to the eutopic endometrium (Table 3, Fig .1C), while the mRNA levels of this isoform were slightly higher in eutopic endometrium samples compared to the control group (Table 3, Fig .1C). However, our data presented no significant differences between these groups (P=0.44). The results generally confirmed that the expression level of *PR-B* significantly differed between groups (P<0.001, Table 3). As shown in Figure 1D, we found significantly lower expression levels of PR-B in endometriotic tissues compared to the controls (P=0.002) and eutopic endometrium tissues (P=0.006, Table 3). Although eutopic endometrium tissues showed low levels of PR-B expression compared with the control samples, there were no significant differences observed among these two groups (P=0.95).

**Fig 2:**
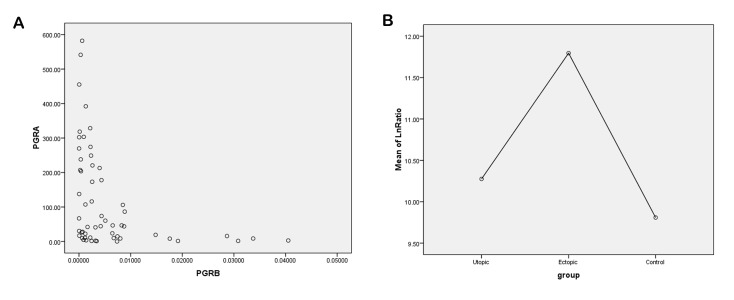
Association of progesterone receptor *(PR)-A* and *PR-B* gene expressions in ovarian endometrioma and endometrium tissues from women with and without endometriosis. **A.** Overexpression of PR-A was associated with a low expression levels of the *PR-B* isoform in the three different groups and **B.** There was a higher *PR-A/PR-B* ratio in endometriutic tissue and eutopic endometrium compared with the control group. Ln; Logarithmic.

**Table 4:** Correlation between mRNA levels of *MMP-2, MMP-9,* and *PR-A/PR-B* ratio in ovarian endometrioma and endometrial tissues from women with and without endometriosis


Different object	Ln*PR-A/PR-B*		
	Endometriotic lesions	Eutopic endometrium	Control endometrium

Ln*MMP-2*	r=0.09	r=0.09	r=-0.19
	P=0.701	P=0.701	P=0.413
Ln*MMP-9*	r=-0.21	r=-0.62^*^	r=0.14
	P=0.365	P=0.003	P=0.542


Pearson test: analysis of correlation between different groups. P values are calculated on logarithmic (Ln)-transformed data. *MMP*; Matrix metalloproteinase, and PR; Progesterone receptor, and r; Spearman’s rho test.

### Association between expression levels of progesterone receptor isoforms *PR-A* and *PR-B* in endometriosis

There was a strong negative correlation between *PR-A* and *PR-B* isoforms in endometriotic lesions. When the PR-A isoform had increased mRNA levels, we found significantly lower levels of PR-B isoform expression and vice versa (r=-0.789, P<0.001, Fig .2A). Similar results were observed in endometrial tissue from women with (r=-0.844, P<0.001) and without endometriosis (r=-0.579, P=0.008).

### *PR-A/PR-B* ratio and its association with *MMP-2* and *MMP-9* expressions in endometriosis

We observed higher *PR-A/PR-B* ratios in both eutopic and endometriutic tissues related to the control group, but this finding was not significant (P>0.05, Table 3, Fig .2B). We were interested to assess the correlation between the mRNA levels of *MMP-2* and the *PR-A/PR-B* ratio in each group. However, our data did not show any significant correlation between overexpression of *MMP-2* and an altered *PR-A/PR-B* ratio in any of the groups (Table 4). We found no significant correlation between the expressions of *MMP-2* and *PR-A* or *PR-B* (P>0.05, data not shown).

Our results indicated that the expression level of *MMP-9* only had a significant relationship to the mRNA levels of the progesterone receptor ratio (*PR-A/PR-B*) in eutopic endometrial tissue (P=0.003, Table 4). On the other hand, we found a significant association among the expression level of *MMP-9* and the *PR-A* isoform in eutopic endometrial tissue (P=0.03). There was no significant relation between the expression levels of *MMP-9* and the *PR-B* isoform in the study groups (P>0.05, data not shown).

## Discussion

Endometriosis develops as a consequence of ectopic implantation of retrograded menstrual tissue, although the mechanisms that underlie this process are unknown (21). Several studies have underlined a correlation between MMPs and the invasive behavior of endometriotic tissues for establishment of endometrial glands and stromal cells at ectopic sites (7). MMPs coordinate general endometrial remodeling through menstrual cycles, which mediates ECM turnover (21). Upregulation and activation of MMPs related to tumor progression have been found in metastatic activity of tumors dependent on MMP synthesis (28). Hence, the expression of MMP enzymes is tightly regulated in normal tissues, because the delicate balance between MMPs and their inhibitors is crucial to preventing excessive matrix destruction (21).

Follicular fluid surrounds the microenvironment of maturing oocytes and has an important role in this process, affecting fertilization and consequent of embryo development (1). The opposed effect of endometriosis on fertilization has been attributed to its impact on the follicular microenvironment, poor oocyte development, and poor embryo formation (4). Studies indirectly suggest that MMP-2 and MMP-9 in follicular fluids have a direct effect on follicular development and rift of the follicular wall (29). A high level of *MMP* expression by the endometriotic tissues can be initiated in the pathogenesis of endometriosis (7). It might be responsible for intrafollicular modifications that result in infertility.

Overexpression of different *MMPs* have been reported in endometriosis and include MMP-1 (30), MMP-2 (18), MMP-9 (20), and MMP-7 (31). The degradation of vascular and epithelial basement membrane components and ECM proteins are mediated by gelatinases (MMP-2 and MMP-9). Gelatinases have been associated with the malignant potential of tumors by increasing tumor invasion and metastasis (32). The role of MMP-2 in endometriosis is debatable. In the current study, we have detected elevated *MMP-2* expression in both ectopic and eutopic tissues of endometriosis patients compared to the normal control group. However, no significant difference in *MMP-2* expression was observed in our groups. Previous studies have reported higher levels of *MMP-2* expression and lower mRNA levels for *TIMP-2* in eutopic tissues of endometriosis patients relative to the endometrium from control groups (33, 34).

This highlights potential changes in MMP activity in endometriotic tissues and suggested improved proteolysis activity, which could play an important role in implantation of this tissue in ectopic sites. In addition, our data showed significantly higher expression levels of MMP-9 in the ectopic versus the eutopic and control endometrial tissues. Several researchers have focused on the role of *MMP-9* in tumor invasion and metastasis (35, 36). The involvement of this proteolytic enzyme in vascular growth and angiogenesis has been previously reported (20). A higher gelatinase activity was found in endometriotic tissues compared to eutopic endometrium in endometriosis (37). Previous investigations have demonstrated higher expression of *MMP-9* in ectopic versus the eutopic endometrium (38). In patients with endometriosis, elevated levels of MMP-9 mRNA in ectopic tissues might play an essential role in endometrial tissue invasion and its ability to be implanted in ectopic sites. High levels of *MMP-2* and *MMP-9* and low levels of *TIMP-1* were related with low production of mature oocytes and subsequent decreased quality of embryos in endometriosis patients who underwent *in vitro* fertilization (IVF) (1). As a result, *MMP-2* and *MMP-9* overexpression have adverse effects on the function of the follicular microenvironment, as well as oocyte and embryo quality. These changes might be the cause of infertility due to endometriosis.

Endometriosis is known as a progesterone resistant disease (23). The ability of progesterone to affect gene expression is reliant on the *PR-A/PR-B* ratio (27). An altered *PR-A/PR-B* ratio modifies progesterone activity due to differential regulation of specific progesterone response target genes that may lead to the progression of endometriosis. Progesterone reduces the expression of pro-inflammatory genes when the *PR-A/PR-B* ratio favors PR-B and increases their expression when the ratio tilts towards the *PR-A* isoform (39, 40). The present study has shown a slightly increased level of *PR-A* expression in eutopic tissues compared to controls. This increased expression was slightly higher in controls compared to ectopic tissues. On the other hand, *PR-B* showed a significantly differential expression pattern between the groups. The results clearly showed a decreased expression level for *PR-B* in endometriotic tissues compared to control and eutopic groups, which can disrupt the *PR-A/PR-B* ratio in ectopic samples. Eutopic tissues also had decreased PR-B expression. Progesterone resistance might account for the existence of the inhibitory PR isoform, *PR-A,* and the lack of the stimulatory isoform, *PR-B*, in endometriotic tissues (23). These results suggested that a decrease in the expression level of *PR-B* and overexpression of *PR-A* could alter this ratio in endometriotic tissues. Following this, the imbalanced ratio could alter progesterone activity related to differential regulation of specific progesterone target genes and improve endometriosis. On the other hand, we have demonstrated an association between overexpression of *PR-A* with low expression of the *PR-B* isoform, particularly in ectopic tissue and the endometria of women with and without endometriosis.

It has been shown that transcriptional regulation of *MMP-2* in the JAr choriocarcinoma cell line is mediated by progesterone treatment with progesterone inhibiting the expression of* MMP-2. MMP-2* expression is mediated through the binding of the primary transcription factor SP4 to the *MMP-2* proximal promoter. Progesterone inhibits *MMP-2* expression by decreasing PR and SP4 binding to the *MMP-2* promoter (24). Progesterone also suppresses TGFβ1-induced stimulation of *MMP-2* through its nuclear hormone receptors in human endometrial stromal cells (22). Therefore, our data imply that observed alteration in *PR-A/PR-B* expression ratio may cause overexpression of MMP-2 in endometriotic tissues. However, our analysis did not show any significant correlation between the high level of *MMP-2* expression and imbalance in *PR-A/PR-B* ratio expression in endometriotic tissues.

In contrast, we have shown, for the first time, a significant association between the expression of *MMP-9* and altered an *PR-A/PR-B* ratio in endometrium (eutopic) tissues of women with endometriosis compared to a normal control group. *MMP-9,* activity in the human endometrium is controlled by estradiol and progesterone (26). This hypothesis can be supported by the fact that progesterone increases the expression level of inhibitor-κBα, a repressor of the NF-кB transcription factor, and inhibits basal and lipopolysaccharide-induced proinflammatory gene expressions via *PR-B,* which are inhibited by *PR-A* (27). NF-κB is involved in the regulation of cytokines and MMP transcription (including *MMP-9*) in the human endometrium. PRs can directly interact with the RelA (p65) subunit of NF-κB, which is necessary to activate NF-кB (26). Thus, an altered *PR-A/PR-B* ratio may impact the expression level of *MMP-9* through the regulation of NF-κB activity, which could be important in the pathogenesis of endometriosis. However, we have not observed any significant correlation between this altered ratio and *MMP-9* expression in ectopic tissues in comparison to the control endometrium samples.

## Conclusion

We sought to assess the correlation between the expression of *MMP-2* and *MMP-9* and the *PR-A/PR-B* ratio in endometriosis. Our data showed a significant negative association between expression levels of *MMP-9* and an altered *PR-A/PR-B* ratio in the eutopic endometrium group compared with the control samples. To our knowledge, there have been few attempts to report these correlations between the MMPs and PR isoforms in endometriosis. It is known that endometriosis affects the follicular microenvironment, oocytes maturity and consequent embryo development. This hypothesis may be correlated further by our observations since overexpression of *MMP-9*, as a consequence of an imbalanced **PR-A/PR-B** ratio in endometriosis, may affect the function of the follicular microenvironment, as well as oocyte and embryo quality, which cause infertility in endometriosis.
